# Pregnancy influences expression of interferon-stimulated genes, progesterone receptor and progesterone-induced blocking factor in ovine thyroid

**DOI:** 10.5713/ab.23.0508

**Published:** 2024-04-25

**Authors:** Jianhua Cao, Shuxin Zhao, Yaqi Zhang, Jiabao Cai, Leying Zhang, Ling Yang

**Affiliations:** 1 School of Life Sciences and Food Engineering, Hebei University of Engineering, Handan 056038, China

**Keywords:** Interferon-stimulated Gene, Progesterone-induced Blocking Factor, Progesterone Receptor, Sheep, Thyroid

## Abstract

**Objective:**

Embryonic interferon-tau (IFNT) and progesterone affect expression of interferon-stimulated genes (ISGs), progesterone receptor (PGR) and progesterone-induced blocking factor (PIBF) in the ovine thyroid.

**Methods:**

Thyroids of ewes were sampled at day 16 of nonpregnancy, days 13, 16, and 25 of pregnancy, and real-time quantitative polymerase chain reaction assay, western blot and immunohistochemistry were used to detect expression of ISGs, PGR, and PIBF.

**Results:**

Free ISG15 protein was undetected, but ISG15 conjugated proteins upregulated at day 16 of pregnancy, and expression levels of ISG15 conjugated proteins, PGR isoform (70 kDa), PIBF, interferon-gamma-inducible protein 10 and myxovirusresistance protein 1 peaked, but expression level of signal transducer and activator of transcription 1 was the lowest at day 16 of pregnancy. In addition, the expression levels of PGR isoform (70 kDa) and signal transducer and activator of transcription 1 (STAT1) decreased, but levels of PGR isoform (43 kDa), 2′,5′-oligoadenylate synthetase, IP-10 and MX1 increased at day 25 of pregnancy comparing with day 16 of the estrous cycle.

**Conclusion:**

Early pregnancy affects expression of ISGs, PGR, and PIBF in maternal thyroid through IFNT and progesterone, which may regulate thyroid autoimmunity and thyroid hormone secretion in ewes.

## INTRODUCTION

The embryonic interferon-tau (IFNT) plays critical roles in preventing luteolysis, and also induces expression of interferon-stimulated genes (ISGs) in corpus luteum (CL) and other tissues in ruminants [1]. Progesterone and IFNT enhances expression of genes in uterine luminal to increase transportation of nutrients into the uterine lumen, and also stimulate expression of mechanistic target of rapamycin for proliferation, migration, and gene expression in the trophectoderm cells during the peri-implantation period of pregnancy in the bovine and ovine [2]. IFNT not only regulates expression of innate immune related-genes in the uterus, but also modulates these genes in peripheral immune cells and other tissues in domestic ruminant animals [3]. It has been reported that expression of ISG15 and ISG15-conjugated proteins in maternal bone marrow, thymus, spleen, lymph nodes and liver upregulates during early pregnancy [4], which is induced by IFNT through an endocrine manner in sheep. In addition, the developing embryo produces IFNT that induces expression of *ISG15* mRNA in endometrium and trophoblast, and also increases abundance of *ISG15* mRNA transcript in intra-hypothalamus and anterior pituitary via blood circulation in ewes [5]. ISG15 is covalently conjugated to target proteins, which regulate a wide range of cellular functions and processes [6].

Thyroxine (T_4_) and triiodothyronine (T_3_) are produced in the thyroid, which regulate key metabolic pathways to control energy balance through the actions in the brain, white fat, brown fat, skeletal muscle, liver, and pancreas in humans, rats and mice [7]. Pregnancy leads to many physiological alterations, and thyroid hormones can cross the placental barrier and evoke biological action in fetal tissues in humans [8]. Thyroid hormone promotes the invasion of extravillous trophoblasts to the decidua, which plays vital roles in the maintenance of early pregnancy in humans and mice [9]. Thyroid hormone supplementation increases total cell counts and decreases proportions of apoptotic cells, and improves bovine embryo development [10]. There are many changes in the function of the thyroid gland during pregnancy, and maternal thyroid dysfunction has adverse effects on the course of pregnancy and fetal development in humans [11]. Progesterone induces gene expression in normal human thyroid follicular cells, and these upregulated genes are implicated in regulating thyroid function and growth [12].

Progesterone modulates expression of gene networks to control development, differentiation, and proliferation of female reproductive tissues via progesterone receptor (PGR) in a tissue/cell type and developmental stage specific manner during the reproductive cycle and pregnancy in humans and mice [13]. PGR is expressed in papillary thyroid carcinoma (PTC), which can be used for the prognosis of patients with PTC in humans [14]. Progesterone binds to PGR on lymphocytes to induce production of progesterone-induced blocking factor (PIBF), which regulates the maternal immune response, and contributes to successful implantation in humans and mice [15]. It has been reported that early pregnancy regulates expression of PGR and PIBF in the maternal immune organs, including thymus, bone marrow, spleen, lymph nodes and liver in the ovine, which is implicated in the maternal immunoregulation [4,16].

It was supposed that early pregnancy had effects on ex pression of ISGs induced by IFNT, and expression of PGR and PIBF induced by progesterone in the maternal thyroid. In this study, we aimed to assess expression of ISGs, including ISG15, signal transducer and activator of transcription 1 (STAT1), 2′,5′-oligoadenylate synthetase (OAS1), myxovirusresistance protein 1 (MX1) and interferon-gamma-inducible protein 10 (IP-10), as well as PGR and PIBF in the ovine thyroid during early pregnancy, which may be beneficial for understanding the adaptation of the thyroid during early pregnancy in ruminants.

## MATERIALS AND METHODS

### Animals and experimental design

All the experimental procedures were approved by the Hebei University of Engineering Animal Care and Use Committee, China (approval no. 2019-017). Nonpregnant Small Tail Han ewes were housed in the farm of Handan Boyuan Animal Husbandry Co. Ltd. in China, and raised under the same conditions with free access to water. Twenty-four ewes were randomly divided into four groups (n = 6 for each group). After detection of estrous behavior (day 0), the ewes were allowed to mate twice at a 12-h interval with intact rams for the pregnant ewes, but the cyclic ewes were not mated with intact rams. Samples of thyroid were obtained from ewes at days 13, 16, and 25 of pregnancy and day 16 of the estrous cycle at the time of slaughter. Pregnancy was confirmed by observing the conceptus in the uterus through dissection. Thyroid samples were cut into small pieces, and immediately fixed in 4% paraformaldehyde for immunohistochemistry analysis, and the remaining portions were frozen in liquid nitrogen for subsequent mRNA and protein analysis.

### RNA extraction and real-time quantitative polymerase chain reaction assay

Total RNA from the thyroid samples was extracted using the TRNzol reagent (Tiangen Biotech Co., Ltd., Beijing, China) according to the manufacturer’s instructions. Approximately 2 μg of total RNA was reverse transcribed into cDNA using a FastQuant RT Kit (Tiangen Biotech Co., Ltd., China). The primer sequences of *ISG15*, *PGR*, *PIBF*, *STAT1*, *OAS1*, *MX1*, *IP-10*, and *GAPDH* genes are shown in Table 1, and *GAPDH* was used as an internal reference to normalize target gene expression. Reactions for the quantification of mRNA by real-time quantitative polymerase chain reaction (RT-qPCR) were performed in a Bio-Rad CFX96 (Bio-Rad, Hercules, CA, USA) using a SuperReal PreMix Plus Kit (Tiangen Biotech Co., Ltd., China). The relative RNA expression value was calculated using the 2^−ΔΔCt^ method. The relative expression value for the group of day 16 of non-pregnancy was set as 1 comparing to other three groups.

### Western blot

Protein samples (10 μg/lane) were separated by sodium dodecyl sulfate polyacrylamide gel electrophoresis and probed by immunoblotting with primary antibodies. The primary antibodies included a mouse anti-ISG15 monoclonal antibody (1:20,000) [4], rabbit anti-PGR polyclonal antibody (Santa Cruz Biotechnology, Inc., Santa Cruz, CA, USA; sc-538), rabbit anti-PIBF polyclonal antibody (Santa Cruz Biotechnology, Inc., USA; sc-99129), a rabbit anti-OAS1 polyclonal antibody (Abcam, Cambridge, UK, ab86343), a mouse anti-IP-10 monoclonal antibody (Santa Cruz Biotechnology, USA; sc-374092), a mouse anti-MX1 monoclonal antibody (Santa Cruz Biotechnology, USA; sc-166412), and a goat anti-STAT1 polyclonal antibody (Abcam, UK; ab230428). The specificity of antibodies was validated, and suitable for sheep. Goat anti-mouse IgG-horseradish peroxidase-conjugated (HRP, Biosharp, Hefei, China; BL001A), anti-goat IgG-HRP (Biosharp, China; BL004A) and anti-rabbit IgG-HRP (Biosharp, China; BL003A) secondary antibodies were used to detect bound proteins. A Pro-light HRP chemiluminescence detection reagent (Tiangen Biotech Co., Ltd., China) was used to visualize the protein bands, which were quantified using Quantity One software (Bio-Rad Laboratories, USA). The values of target proteins were normalized to GAPDH protein using an anti-GAPDH antibody (Santa Cruz Biotechnology, USA; sc-20357).

### Immunohistochemistry analysis

The fixed thyroid samples were embedded in paraffin, and cut into 5-μm-thick sections. After deparaffinized and hydrated in graded ethanol series, some sections were stained by hematoxylin and eosin. Other sections were quenched endogenous peroxidase activity with 3% hydrogen peroxide, and blocked in 5% normal goat serum. After incubation with the ISG15 monoclonal antibody (1:2,000), or rabbit anti-PGR polyclonal antibody (1:100), the sections were washed three times with PBS. For negative controls, non-immune goat serum was used in place of the primary antibody, and absorption test was also performed to verify the specificities of the primary antibodies using the target proteins, and no positive signal was detected. Anti-mouse IgG-HRP (Biosharp, China; BL001A), or anti-rabbit IgG-HRP (Biosharp, China; BL003A) secondary antibodies were used to detect target proteins. Nuclei were counterstained with hematoxylin, and the antibody binding sites in the thyroid tissue were detected using a DAB kit (Tiangen Biotech Co., Ltd., China). Images were observed using a light microscope (Nikon Eclipse E800, Tokyo, Japan) with digital camera DP12, and the intensity of staining and density of staining cells were analyzed in a blinded fashion as described previously [4].

### Statistical analysis

All data were evaluated as mean±standard error of the mean. The data for relative expression levels of *ISG15*, *PGR*, *PIBF*, *STAT1*, *OAS1*, *MX1*, and *IP-10* mRNA and proteins were analyzed using the general linear model procedure (SAS Inst. Inc., Cary, NC, USA). Multiple testing correction was performed using Bonferroni post-test correction to adjust a significant p-value. Statistical significance is defined when p values are less than 0.05.

## RESULTS

### Relative values of *ISG15*, *PGR*, *PIBF*, *STAT1*, *OAS1*, *MX1*, and *IP-10* mRNA in the thyroid

**Figure 1:**
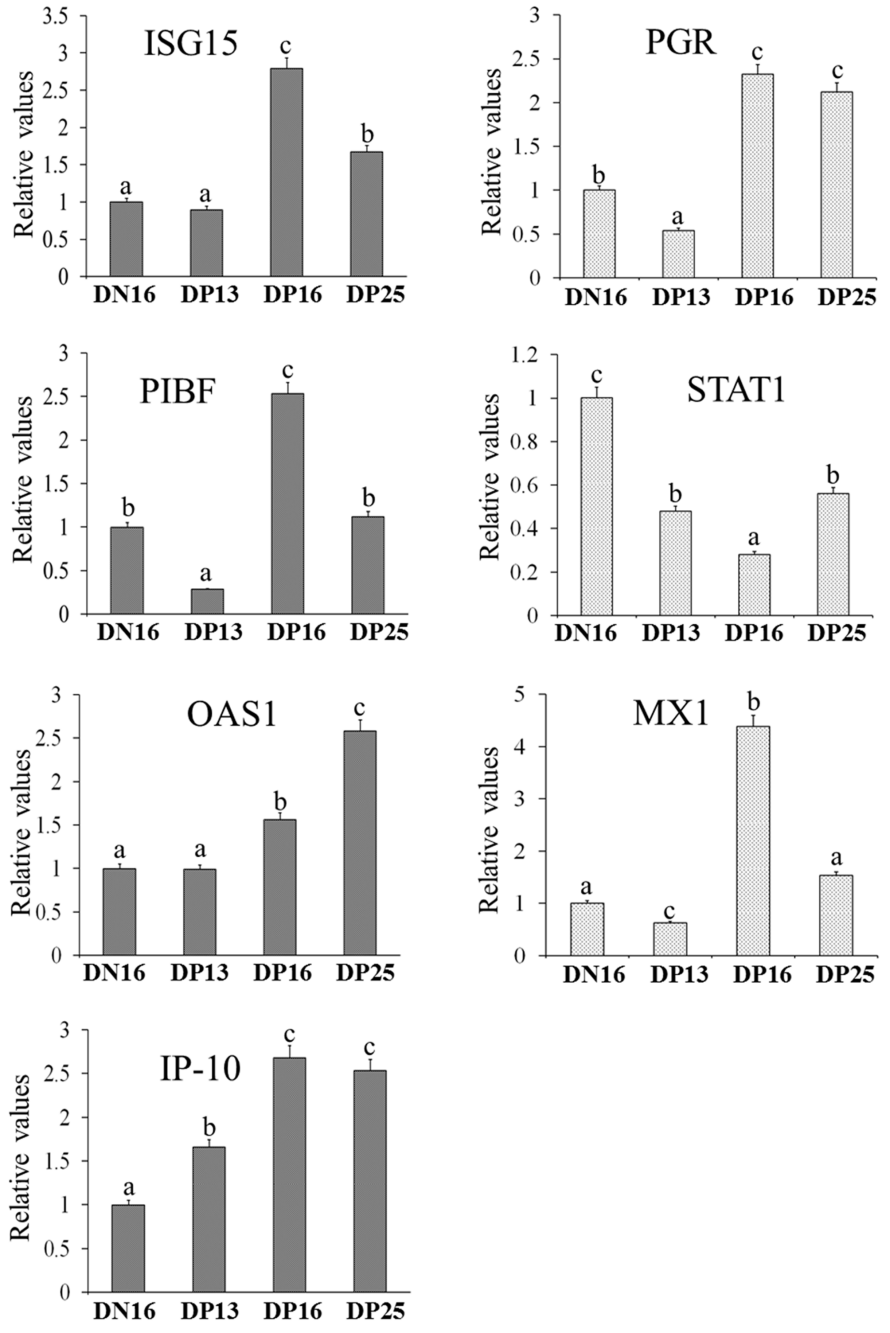
Relative expression value of *ISG15*, *PGR*, *STAT1*, *OAS1*, *MX1* and *IP-10* mRNA in thyroids. DN16, day 16 of non-pregnancy; DP13, day 13 of pregnancy; DP16, day 16 of pregnancy; DP25, day 25 of pregnancy. ^a–c^ Significant difference (p<0.05) was indicated by different letters.

Figure 1 shows that the relative values of *ISG15*, *PGR*, *OAS1*, and *IP-10* mRNA upregulated at days 16 and 25 of pregnancy (p<0.05), but the values of *PGR* and *PIBF* were the lowest at day 13 of pregnancy among the four groups (p<0.05). In addition, the values of *ISG15*, *PIBF*, and *MX1* mRNA were the highest, but the value of *STAT1* mRNA was the lowest at day 16 of pregnancy among the four groups (p<0.05). However, early pregnancy inhibited the expression of *STAT1* mRNA (p<0.05), and there were no statistical differences in the relative levels of *PIBF* and *MX1* mRNA between nonpregnancy and day 25 of pregnancy (p>0.05).

### Expression of ISG15, PGR, STAT1, OAS1, MX1, and IP-10 proteins in the thyroid

**Figure 2:**
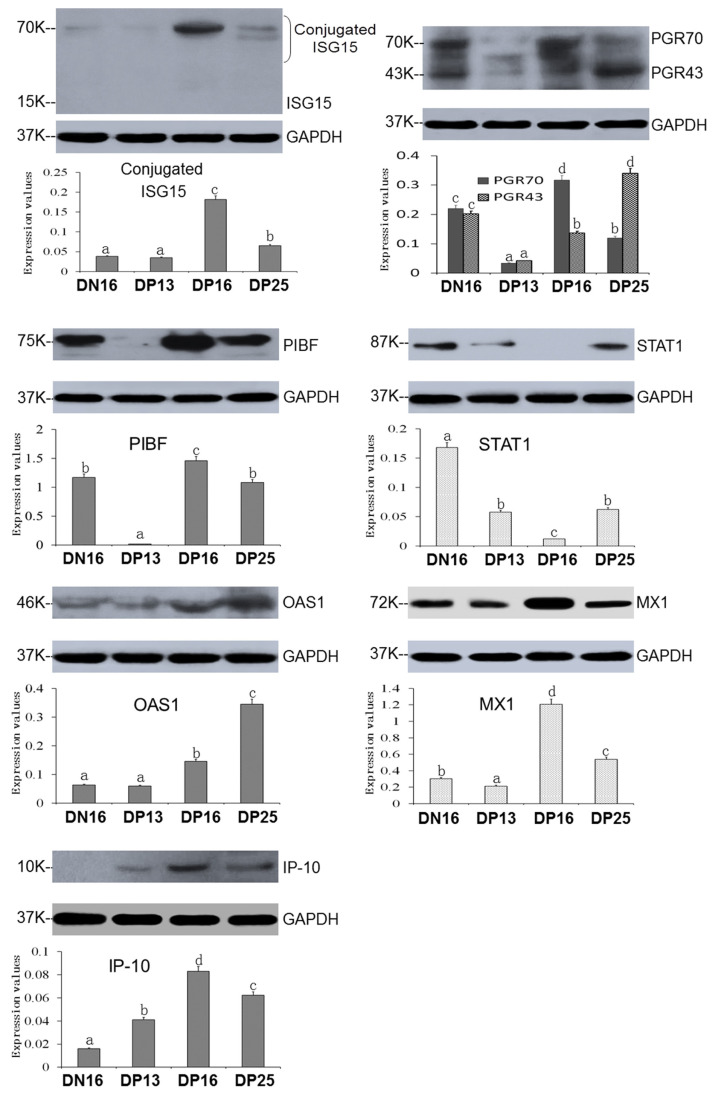
Expression of ISG15, PGR, STAT1, OAS1, MX1 and IP-10 proteins in thyriods. DN16, day 16 of non-pregnancy; DP13, day 13 of pregnancy; DP16, day 16 of pregnancy; DP25, day 25 of pregnancy. Immunoreactive proteins greater than 30 kDa were deemed ISG15 conjugated proteins. PGR70, isoform of PGR with molecular weight of 70 kDa; PGR43, isoform of PGR with molecular weight of 43 kDa. ^a–d^ Significant differences (p<0.05) are indicated by different superscript letters.

Figure 2 reveals that free ISG15 protein was undetected, but expression level of conjugated ISG15 proteins were the highest at day 16 of pregnancy. In addition, conjugated ISG15 protein was almost undetected at day 13 of pregnancy and day 16 of nonpregnancy. Expression levels of PGR70, PGR43, PIBF, and MX1 proteins were the lowest at day 13 of pregnancy (p<0.05), but levels of PGR70, PIBF, MX1, and IP-10 proteins were the highest at day 16 of pregnancy among the four groups (p<0.05). Furthermore, expression level of PGR43 was the highest at day 25 of pregnancy among the four groups (p<0.05).

There was a decrease in expression of STAT1 protein during early pregnancy (p<0.05), and STAT1 protein was almost undetected at day 16 of pregnancy. Early pregnancy enhanced expression of MX1, OAS1, and IP-10 proteins at days 16 and 25 of pregnancy (p<0.05), with a peak at day 25 of pregnancy for OAS1 protein among the four groups (p<0.05). Furthermore, IP-10 protein was almost undetected at day 16 of nonpregnancy (Figure 2).

### Immunohistochemistry for ISG15 and PGR proteins in the thyroid

**Figure 3:**
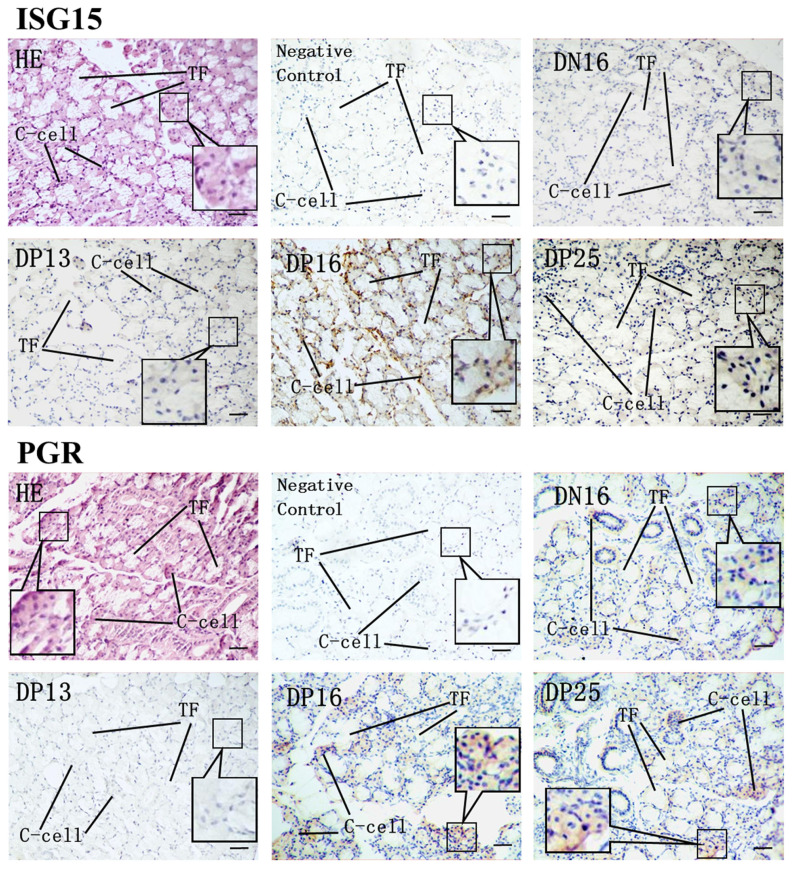
Representative immunohistochemical localization of ISG15 and PGR in ovine thyroids collected on day 16 of the estrous cycle, and days 13, 16, and 25 of pregnancy. The thyroid follicle (TF) containing colloid in their lumina are lined predominantly parafollicluar cell, also known as thyroid medullary cells (C-cells). The immunohistochemistry does not distinguish the different isoforms of PGR in the thyroid tissue sections. HE, haematoxylin-eosin; DN16, day 16 of pregnancy; DP13, day 13 of pregnancy; DP16, day 16 of pregnancy; DP25, day 25 of pregnancy. Bar = 50 μm.

Immunohistochemistry analysis confirmed that ISG15-immunoreactive cells were parafollicluar cells, also known as thyroid medullary cells (C-cells) (Figure 3), and staining for ISG15 and/conjugated proteins was the strongest in thyroids from day 16 of pregnancy compared with other stages. The immunohistochemistry also did not distinguish the different isoforms of PGR in the thyroid tissue sections. The districts of immunostaining for PGR proteins were limited to C-cells, and staining for PGRs was the weakest in thyroids from day 13 of pregnancy compared with other stages (Figure 3).

## DISCUSSION

In this study, the ISG15-conjugated protein increased at day 16 of pregnancy, and ISG15 conjugated proteins were limited to the C-cells. ISG15 is an ubiquitin like protein, and plays a direct role in mitigating DNA replication stress and promoting genomic stability via ISG15 conjugation (ISGylation) [17]. ISG15 is produced by interferon stimulated gene 15, and ISG15 and ISGylation have profound impact on protein translation, exosome secretion, cytokine secretion and immune modulation [18]. The level of *ISG15* mRNA in peripheral blood mononuclear cells (PBMCs) and polymorphonuclear leukocytes is higher in pregnant buffalo cows than non-pregnant cows during the peri-implantation period [19]. IFNT secreted by the conceptus acts on PBMCs to enhance expression of *ISG15* mRNA and consentation of serum ISG15 protein, and activate ISGlyation enzymes *UBE1L* and *UBCH8* on day 18 of pregnancy in cows [20,21]. IFNT upregulates expression of *ISG15* mRNA transcript in trophoblast and endometrium, hypothalamus, anterior pituitary, and corpus luteum by blood circulation during early pregnancy in sheep [5]. In addition, ISG15 and ISG15 conjugated proteins increase in maternal bone marrow, thymus, spleen, lymph nodes and liver [4], which are related to maternal immunoregulation during early pregnancy in ewes. C-cells are involved in synthesis of thyroid hormones thyroxine, triiodothyronine and calcitonin [22]. Therefore, the upregulation of ISG15 conjugated proteins in the C-cells may be related with the IFNT from the conceptus, and associated with the modulation of the function of the maternal thyroid during pregnancy.

It was shown in this study that expression of STAT1 de clined during early pregnancy. STAT1 is a nuclear transcription factor, and plays essential roles in regulating cell cycle, cell survival and immune response [23]. Early pregnancy upregulates expression of STAT1 on days 13 and 16 of gestation, but downregulates STAT1 expression on day 25 of pregnancy in maternal thymus compared with the nonpregnant controls in sheep [24]. In addition, there is a downregulation of STAT1 protein in the superficial glandular epithelium and subepithelial stromal cells intense on day 15 of pregnancy, but an upregulation on day 18 of pregnancy comparing with day 5 of pregnancy in the goat endometrium [25]. Therefoore, downregulation of STAT1 during early pregnancy may be related to the adaptation of thyroid gland.

Our data showed that expression of OAS1, MX1 and IP-10 was increased at early stage of pregnancy. OAS can enhance proinflammatory cytokine secretion through activating the RNA cleavage pathway [26]. There is an upregulation of MX1 mRNA in peripheral blood leukocytes on day 21 of gestation, which can be used to predict the gestational status in cows [27]. Early pregnancy enhances expression of MX1 and OAS1 in neutrophils, which is related to immunoregulation during the peri-implantation period in dairy cows [28]. There is an upregulation of OAS1 and IP-10 in maternal spleen and liver [29], and MX1 in maternal lymph nodes and thymus [24,30] during early pregnancy in sheep, which are induced by IFNT, and associated with immunoregulation of pregnancy in ewes. Central nervous system-native myeloid cells (CNS-myeloids) modulates brain immunity, and upregulation of IP-10 in CNS-myeloids is related to immune-suppression in the brain [31]. Therefore, the upregulation of OAS1, MX1 and IP-10 at early stage of pregnancy may be associated with the immunoregulation of maternal thyroid gland.

Our results revealed that expression level of isoform PGR70 decreased, but level of isoform PGR43 increased at day 25 of pregnancy, and PGRs were located in the C-cells. During the reproductive cycle and pregnancy, PGR can regulate target gene expression via tissue/cell-type-specific PGR signaling pathways to control developmental processes, proliferation and differentiation in an endocrine manner in humans and mice [13]. The remodeling of the cervix is controlled by progesterone via PGR signaling, which is essential for pregnancy maintenance during normal pregnancy in humans, rats and mice [32]. PGR isoforms are collectively involved in physiological adaption of the uterus through their distinct and common downstream target genes in the endometrium during early pregnancy and parturition in humans, rats and mice [33]. Progesterone regulates gonadotropin secretion, preparation of the endometrium for implantation, and maintains pregnancy via modulating expression of PGR isoforms in humans [34]. Progesterone exerts direct effects on thyroid cells to regulate gene expression via PGR, which is related to thyroid function and growth in humans [12]. Early pregnancy modulates expression of PGR isoforms in maternal thymus, bone marrow, spleen, lymph nodes and liver during early pregnancy, which are related to the adaptation of maternal immune organs in ewes [4,16]. C-cells produce thyroid hormones and calcitonin [22]. Therefore, the changes in expression of PGRs in the C-cells may be related to the adaptation of thyroid function to pregnancy.

In the present study, expression level of PIBF was the lowest at day 13 of pregnancy, but was the highest at day 16 of pregnancy. PIBF is an immunomodulatory protein, and plays a key role for cells in escaping immune surveillance in humans and mice [35]. There are significantly higher serum PIBF levels in fertile females which are favorable for endometrial adaptation, embryonic implantation and development during pregnancy in humans than those with unexplained-infertility [36]. Progesterone and PIBF concentrations increase with advancing trimester in pregnant women, which play an essential roles in maintaining normal pregnancy [37]. PIBF is expressed in peripheral lymphocytes and other cell types, which mediates the immunological actions of progesterone, and induces the T helper 2 (Th2) dominant immune responses during normal pregnancy in humans and mice [38]. PIBF acts in favor of a Th2-type immunity, which is necessary for gross alterations in endocrine functions owing to the semi-allogeneic conceptus, and supporting a healthy pregnancy outcome in humans, rats and mice [39]. Expression levels of PIBF isoforms in the maternal thymus, bone marrow, spleen, lymph nodes and liver are regulated by early pregnancy, which are associated with the immune tolerance of maternal immune system in ewes [4,16]. In the present study, expression level of PIBF was the lowest at day 13 of pregnancy, but was the highest at day 16 of pregnancy, which may have effects on the endocrine function of the thyroid during early pregnancy in ewes.

**Figure 4:**
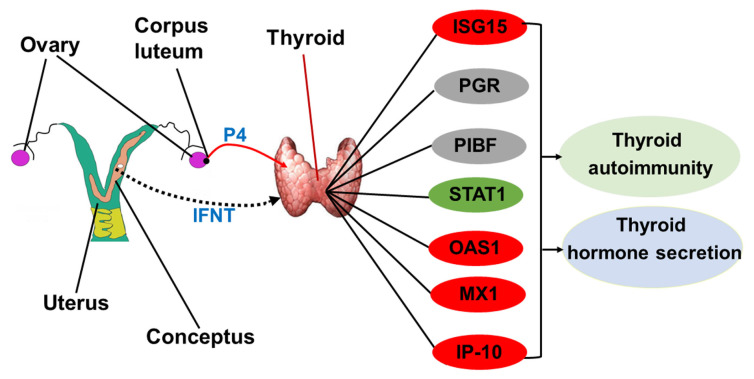
Sketch of interferon-stimulated genes, progesterone receptor and progesterone-induced blocking factor in ovine thyroid during early pregnancy in sheep. Early pregnancy signal (IFNT) from conceptus and progesterone (P4) from corpus luteum exert effects on the thyroid, which modulate expression of interferon-stimulated genes, including *ISG15*, *STAT1*, *OAS1*, *MX1*, and *IP-10*, as well as progesterone receptor (*PGR*) and progesterone-induced blocking factor (*PIBF*), which contributes to adaptation and immunoregulation of thyroid during early pregnancy in ewes. Red, increase; green, decrease; gray, changed.

In this study, IFNT from the conceptus and progesterone from CL have effects on the maternal thyroid through an endocrine manner, which lead to changes in expression of PGR and PIBF, increases in expression of ISG15, OAS1, IP-10, and MX1, but decreases in expression of STAT1. The modulation of ISGs, PGR, and PIBF is related to thyroid autoimmunity, the synthesis and release of T_4_ and T_3_. The thyroid is responsible for synthesis and release of T_4_ and T_3_, which has important effects on numerous tissues, including liver, kidney, thyroid, skin, and placenta in humans [40]. Therefore, early pregnancy exerts effects on maternal thyroid to regulate thyroid autoimmunity and hormone secretion in ewes (Figure 4).

## CONCLUSION

Early pregnancy stimulated expression of ISG15-conjugated protein at day 16 of pregnancy, and expression of PGR and PIBF was modulated. In addition, expression of OAS1, IP-10, and MX1 upregulated, but expression of STAT1 downregulated during early pregnancy. This paper reports for the first time that expression of ISGs, PGR, and PIBF in the maternal thyroid was modulated during early pregnancy, which may be associated with thyroid autoimmunity and secretory function.

## Figures and Tables

**Table 1:** The primer sequence for quantitative real-time polymerase chain reaction

Gene	Primer	Sequence	Size (bp)
*ISG15*	Forward	CATCCTGGTGAGGAACGACAA	186
	Reverse	AAAGACAGCCAGAACTGGTCC	
*PGR*	Forward	CAACAGCAAACCTGATACCT	183
	Reverse	CCATCCTAGTCCAAATACCATT	
*PIBF*	Forward	CCAGGCAGCTAATTGAACGG	189
	Reverse	GGGCTAGTACCTGCTTCTGG	
*STAT1*	Forward	GTGGCGGAGAGTCTGCAGCA	190
	Reverse	GGTGAGTTGGCATGCAGGGC	
*OAS1*	Forward	AGCCTTCCTGAAGAGTCGTCCTAC	88
	Reverse	TCCAAGCTGCTCCTTACACAGTTG	
*MX1*	Forward	CCACCACCGACAGCTCCCCT	147
	Reverse	GCAGGTGTGGGCGTGAAGCA	
*IP-10*	Forward	TCTAGGAACACACGCTGCAC	108
	Reverse	GACACGTGGGCAGGATTGAC	
*GAPDH*	Forward	GGGTCATCATCTCTGCACCT	176
	Reverse	GGTCATAAGTCCCTCCACGA	
